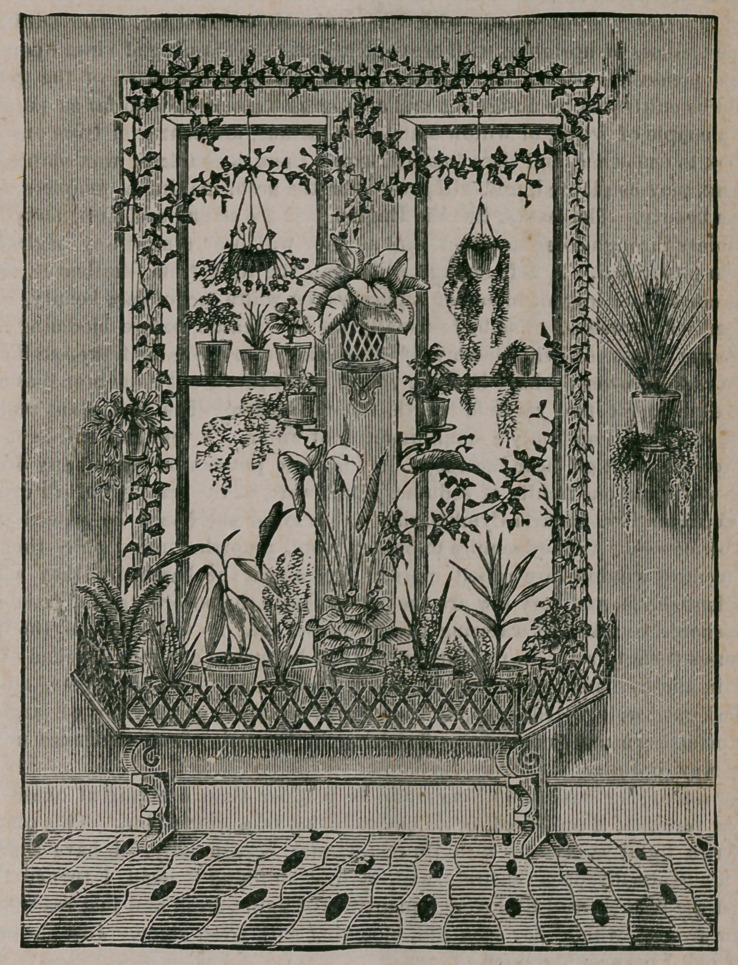# Household

**Published:** 1888-01

**Authors:** 


### HOUSEHOLD.

Window Gardening.—There is in window gardening one advantage not found in using pots alone, as in parlor gardening. Window-boxes may here be used, and less care will be required, and so a greater variety of plants may be cared for in small space. As shown in the cut, the window garden consists chiefly of pots set upon brackets and shelves, making a very ornaméntal appearance; but a box or boxes may be used alone, or with pots and vines trained up the windows or upon the walls. Hanging baskets make a fine addition, especially in the absence of brackets.

The window box may be a simple wooden box of the shape desired, or more elaborate and expensive, and made of pottery ware or lined with zinc as the taste and means dictate. A box of the length of the window-sill, or two made to fit the sill for convenience in handling, covered inside with pitch, or they may be used plain. The outside may be ornamented with coarse bark or simply painted. The earth should be light and porous, and with coarse gravel or charcoal in the bottom. If preferred, there may be small boxes placed upon an old table, or on a stand purposely constructed, with one stair above another. The addition of simple trellises or uprights, with cross-pieces and the training of vines upon them, gives a pleasant variety, but whatever may be said, the taste of the gardener should be cultivated by designing and arranging these details so far as possible.

If a couple of wires, or willow stands, or rattans, are bent over from one end of the box to the other, and slats fastened across them, a couple of wax plants, *Hoya carnosa,* may be planted, and will grow over the arch, making a complete mass of waxen beauty. The leaves, like the English ivy, are strong and firm, never falling off unless through some accident, and as they will bear almost any neglect, they seldom lose a leaf. A soft sponge will clean the dust from the leaves, and the great clusters of fragant bloom of a pure waxen white, are of rare beauty, remaining perfect for weeks. The buds usually set the season before blooming, and if the flower is not cut, will bloom year after year from the same flower-stalk. It is also a rapid grower ; I have had one young plant throw out four branches or runners, each about two yards in length, in one season. The wax plant requires a light, sandy soil, and only a small quantity of water. A very rich soil kept moist, will rot the plant off near the root. In the same pot can be placed any drooping vine, the numerous family of sedums, moneywort, or the prettiest of all droopers, the Coliseum Ivy, Linaria Cymbalaria. On a lower shelf may be placed pansies and primroses. As they require to be kept cool, a north window suits them best, but as most windows are raised more or less above the baseboard, they will find sufficient shade, and verv few are the house plants that afford greater satisfaction. Care should be taken in watering the primroses that no water fall on the bud, as it causes them to rot. In the spring they can be turned out into the shady border, and in another autumn should be divided and put into small pots, and kept shaded until well rooted, then, as they grow, repotted, using a pot of larger size. The primrose usually commences flowering in December, and continues until the next May or June.

For a hanging basket, nothing could be prettier than a clump of oxalis bulbs, mixed varieties, if preferred, or an entire basket of a single kind. This plant also prefers a light, sandy soil with liquid manure, occasionally. I have sometimes had my oxalis disappear entirely, and, at first thinking it dead, I turned the soil out of the pot, where I discovered quantities of bulbs, fresh and just ready to start a new growth, which they soon did, blooming with fresh vigor. Just the cause of the freak I never understood.

For the shelf in front, a tall, trellised fuschia for center piece, with foliage plants on either side, will make a brilliant showing, and after filling the box with geraniums or other shrubbery plants, set German ivies, or any other drooping vines, along the edge to cover the front of the box, and twine about the rustic supports. No window will be perfect without pots of English ivy on either side to arch the window. Too much cannot be said in praise of this hardy plant for indoor use. Growing either in the shade or in the sunlight, bearing almost any change of temperature to which a living room is subjected, it still thrives and goes on its quiet way to beauty and perfection.

Madeira bulbs planted in boxes and hung behind pictures, will send out dainty runners and entirely wreathe the cords and frames; although preferring strong sun and rich earth, yet they will do very well on the most scanty fare. And the common ground ivy—the Jill-go-over-the-ground—of our childhood's kitchen garden, makes a very airy, delicate basket plant. The sweet alyssum of our mother's flower borders is also utilized, and this, like the mignonette, should be planted in the pot where it is expected to bloom, as transplanting after the profuse blooming of summer they are sure to die.

All plants, as well as people, require fresh air, and their leaves, which serve for lungs, should be kept as free from dust as possible. A fine rose watering-pot will aid in cleaning the leaves and leaf stalks, always showering the plants in preference to pouring a quantity of water about the roots, which necessitates a strip of oilcloth underneath the plants, that can be readily wiped up, giving the plants proper care without injuring the carpets.

A steady temperature of from sixty to seventy degrees by day, and down to fifty at night, is best adapted to the requirements of most plants grown in common windows.

Lilies should always be re-potted in autumn, and kept in a cool frame or other cool, moist place all winter. They begin to grow in the spring, and, as soon as the weather will permit, plunge the pots in ashes or sphagnum, where they can be kept from drying out during the summer. Success in growing lillies in pots may be achieved when a total failure would result from planting them in beds.

Begonias.—"If obliged to confine myself to one class of plants for window culture, I would," writes a correspondent of the *Horticultural Times*, "select the flowering begonias. They bear dry heat and occasional neglect as well as any, and are not liable to the attacks of insects, while the number and variety of species are large. Next to begonias I would place geraniums. Everyone knows how endless is the variety of shades and forms of these beautiful flowers. Then the scented, the silver-leaved, the bronze, the ivy-leaved, the tri-colored,—a charming array. One can have a gay window without any flowers at all. The list of desirable plants is almost endless."

Large Plants in Small Pots.—It is well known that geraniums will bloom earlier and better in small pots than large ones, and the small pots are much the more convenient for them and other plants in windows, where large ones are unsightly, and take up much room. It is an interesting experiment to keep a plant in continual and perfect bloom all summer, in a pot which, compared with its own bulk, is so diminutive that it is a wonder to the uninitiated how such a grand dome of leaves and flowers could possibly have developed from such a thimbleful of soil. The secret is in using once or twice a week, after the roots have filled the soil, dilute liquid manure, which is immediately appropriated if the roots have not been left so long without food or drink as to be starved and shrunken. Soot is an excellent article for use in this way.

## Figures and Tables

**Figure f1:**